# Human gut epithelium features recapitulated in MINERVA 2.0 millifluidic organ-on-a-chip device

**DOI:** 10.1063/5.0144862

**Published:** 2023-09-19

**Authors:** Francesca Donnaloja, Luca Izzo, Marzia Campanile, Simone Perottoni, Lucia Boeri, Francesca Fanizza, Lorenzo Sardelli, Emanuela Jacchetti, Manuela T. Raimondi, Laura Di Rito, Ilaria Craparotta, Marco Bolis, Carmen Giordano, Diego Albani

**Affiliations:** 1 Department of Chemistry, Materials and Chemical Engineering ‘Giulio Natta,’ Politecnico di Milano, Milan, Italy; 2 Department of Oncology, Computational Oncology Unit, Istituto di Ricerche Farmacologiche Mario Negri IRCCS, Milan, Italy; 3 Department of Neuroscience, Istituto di Ricerche Farmacologiche Mario Negri IRCCS, Milan, Italy

## Abstract

We developed an innovative millifluidic organ-on-a-chip device, named MINERVA 2.0, that is optically accessible and suitable to serial connection. In the present work, we evaluated MINERVA 2.0 as millifluidic gut epithelium-on-a-chip by using computational modeling and biological assessment. We also tested MINERVA 2.0 in a serially connected configuration prodromal to address the complexity of multiorgan interaction. Once cultured under perfusion in our device, human gut immortalized Caco-2 epithelial cells were able to survive at least up to 7 days and form a three-dimensional layer with detectable tight junctions (occludin and zonulin-1 positive). Functional layer development was supported by measurable trans-epithelial resistance and FITC-dextran permeability regulation, together with mucin-2 expression. The dynamic culturing led to a specific transcriptomic profile, assessed by RNASeq, with a total of 524 dysregulated transcripts (191 upregulated and 333 downregulated) between static and dynamic condition. Overall, the collected results suggest that our gut-on-a-chip millifluidic model displays key gut epithelium features and, thanks to its modular design, may be the basis to build a customizable multiorgan-on-a-chip platform.

## INTRODUCTION

Over the last decades, the gut raised a strong interest in the health domain due to its link with the pathophysiology of other systems such as the cardiovascular and the central nervous one.^1–4^ Actually, gut epithelial cells, together with their resident micro-organisms (microbiota), are involved in many functions, including the modulation of the host immune system. Being also an active physiological barrier that separates the intestinal lumen from the systemic circulation, gut epithelium is the first line of adsorption of many drugs or nutraceuticals.^5–7^ Under pathological circumstances, such as the “leaky-gut” condition, gut permeability significantly increases, causing microbial biologically active molecules to pass into the blood vessels contributing to several disorders, including neurodegeneration.^6–9^ This is the functional basis of the microbiota-gut-brain-axis (MGBA), a recently proposed bidirectional connection between our intestinal microbiota and the brain. The MGBA is an intriguing concept as it features complex networks of multiple biological systems that may link the gut microbiota metabolic activity with neuro-related pathologies such as Alzheimer's (AD) or Parkinson's disease (PD).^9–13^

The investigation of the MGBA is challenging and many researchers have explored innovative *in vitro* engineered models to recall the complexity of human organs featured in the MGBA. This goal can be achieved also by organ-on-a-chip (OoC) devices, suitable to assess the mechanisms involved in human tissue/organs interaction and useful for pre-clinical research. In this context, the “MINERVA” project aims at developing a multi-OoC platform that will recapitulate the main players involved in the MGBA crosstalk: the microbiota, the gut epithelium, the immune system, the blood–brain barrier, and the brain.^9–11,13–16^ To this purpose, we designed an innovative millifluidic OoC device, named MINERVA 2.0, to address some limitations of the so-far available OoCs. Indeed, most of the available OoC microfluidic devices work with limited quantities of cells and culture media, preventing the use of some biological/biochemical assays for cell characterization. Furthermore, many of them need complex production protocols that requires expensive equipment and sophisticated cleanroom facilities,^17^ may be not optically accessible,^17^ or need complicated handling requirements, which greatly affect usability in routine academic and industrial laboratories.^18^ Finally, often current devices cannot be easily connected to form a platform suitable to address multiorgan crosstalk.^19–23^ MINERVA 2.0 not only serves as the basic unit of MGBA-modeling MINERVA platform but may be also a starting point of different customizable multiorgan platforms.^9–11,13–16^ Its design involves inlet and outlet perfusion channels equipped with commercial hydraulic connectors that allow easy connection.^24–26^ In addition, our device is user-friendly, cost-affordable, optically accessible, and compatible with many assays based on low-to-medium biologic material input. Aiming at developing the whole MINERVA platform, we tailored MINERVA 2.0 to develop a physiological gut model, the first human compartment of the MGBA.^27^ Several microfluidic *in vitro* “gut-on-a-chips” have been so far developed. Evidence from the literature suggests that under perfusion the intestinal microarchitecture and cell differentiation might be managed by controlling the basolateral fluid flow applied to the cells.^6,19,22,28–31^ Furthermore, in these systems, the assessment of trans-epithelial electrical resistance (TEER) and FITC dextran permeability assays supported OoC suitability in reproducing *in vivo* some key intestinal features, such as tight-junctions and solute regulated transport, as well as mucin production.^20–23,32,33^

We here validate MINERVA 2.0 as suitable gut-on-a-chip device by involving computational modeling and biological assessment with a human gut immortalized Caco-2 epithelial cell-based model. We also verified MINERVA 2.0 performance when serially connected, a prodromal step to address the complexity of multiorgan interaction as in the MGBA modeling.

## RESULTS

### MINERVA 2.0 device description

**FIG. 1.**
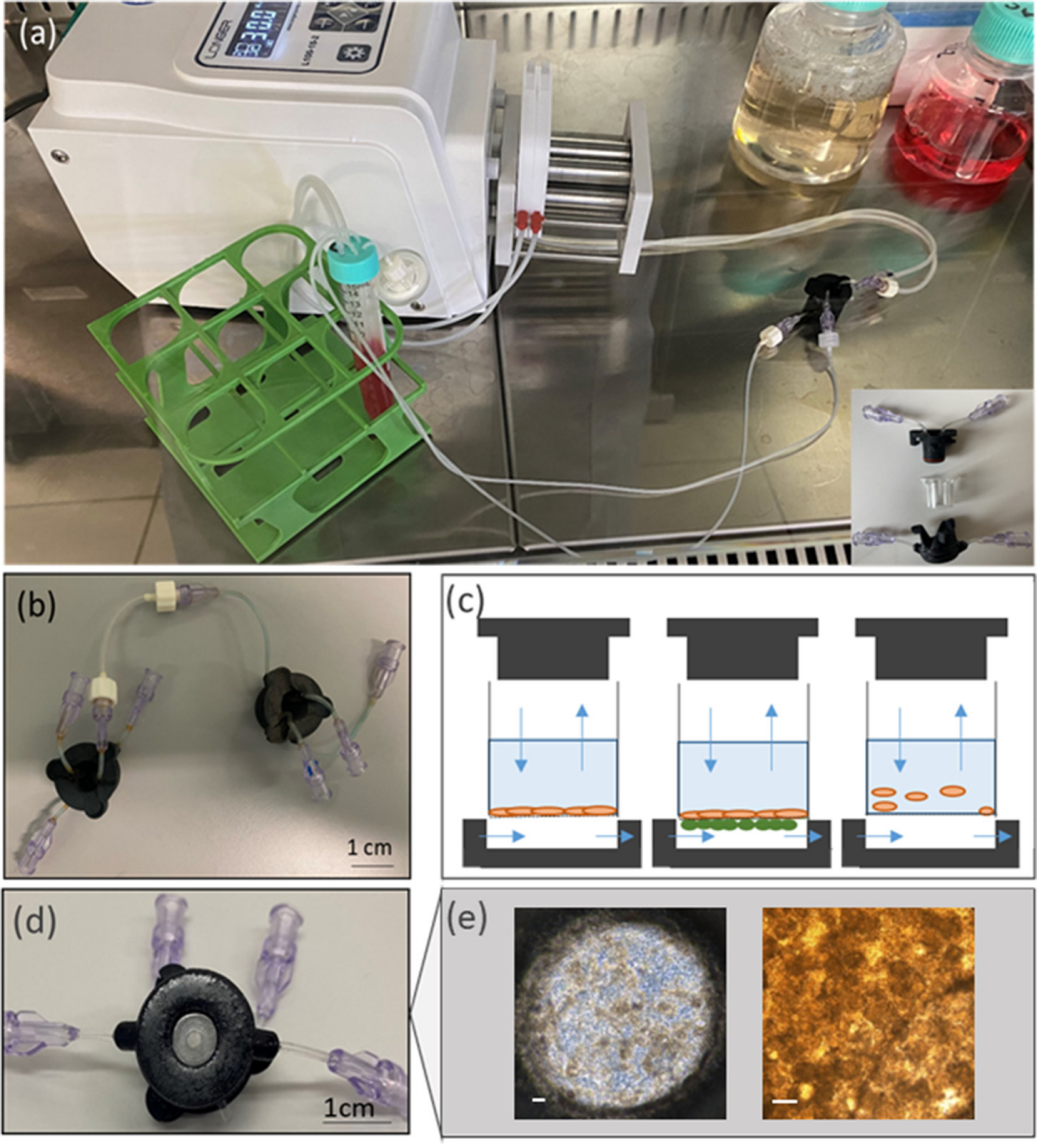
MINERVA device. (a) View of the MINERVA 2.0 setup. On the bottom right is an exploded representation of the MINERVA 2.0 assembly, consisting of apical and basal components enclosing the Transwell-like insert. (b) The Luer-lock connection guarantees the MINERVA possibility to be connected to other MINERVA devices. (c) Sketch of MINERVA 2.0 application. Single cell layer in adhesion in the insert (left), double cell layer across the insert membrane (center), cells in suspension in the insert (right). Blue arrows correspond to flow direction according to the design of the inlet and outlet. (d) Bottom view of MINERVA 2.0 with a transparent glass slide in the center for optical access. (e) The Caco-2 cell layer inside the device. The scale bar corresponds to 100 *μ*m.

MINERVA 2.0 (Fig. 1) was designed starting from the know-how produced for our previously developed OoC device.^34^ Its basic unit uses a nylon 3D printable structure consisting of two components coupled manually by a snap-fit closure system [Fig. 1(a)] that sandwiches a 12-well Transwell-like insert (Greiner Bio-One) with a PET membrane having pore diameter of 0.4 *μ*m and density of 2 × 10^6^ pores/cm^2^. Once assembled, the MINERVA 2.0 provides two connected culture hemi-chambers, one apical and the other one basal, both 0.5 mm high [Fig. 1(c)].

A double O-ring configuration guarantees a reliable seal. On both apical and basal MINERVA 2.0 components, a transparent glass slide is mounted for optical or confocal microscope access [Figs. 1(d) and 1(e)]. Independent perfusion is possible in the two hemi-chambers in both concurrent and countercurrent configuration.

MINERVA 2.0 is equipped with Luer-lock connectors attached to millifluidic channels with diameter 0.5–1 mm allowing for the assembly of a multiorgan platform configuration [Fig.1(b)].

### Computational model to implement MINERVA 2.0 device perfusion

**FIG. 2.**
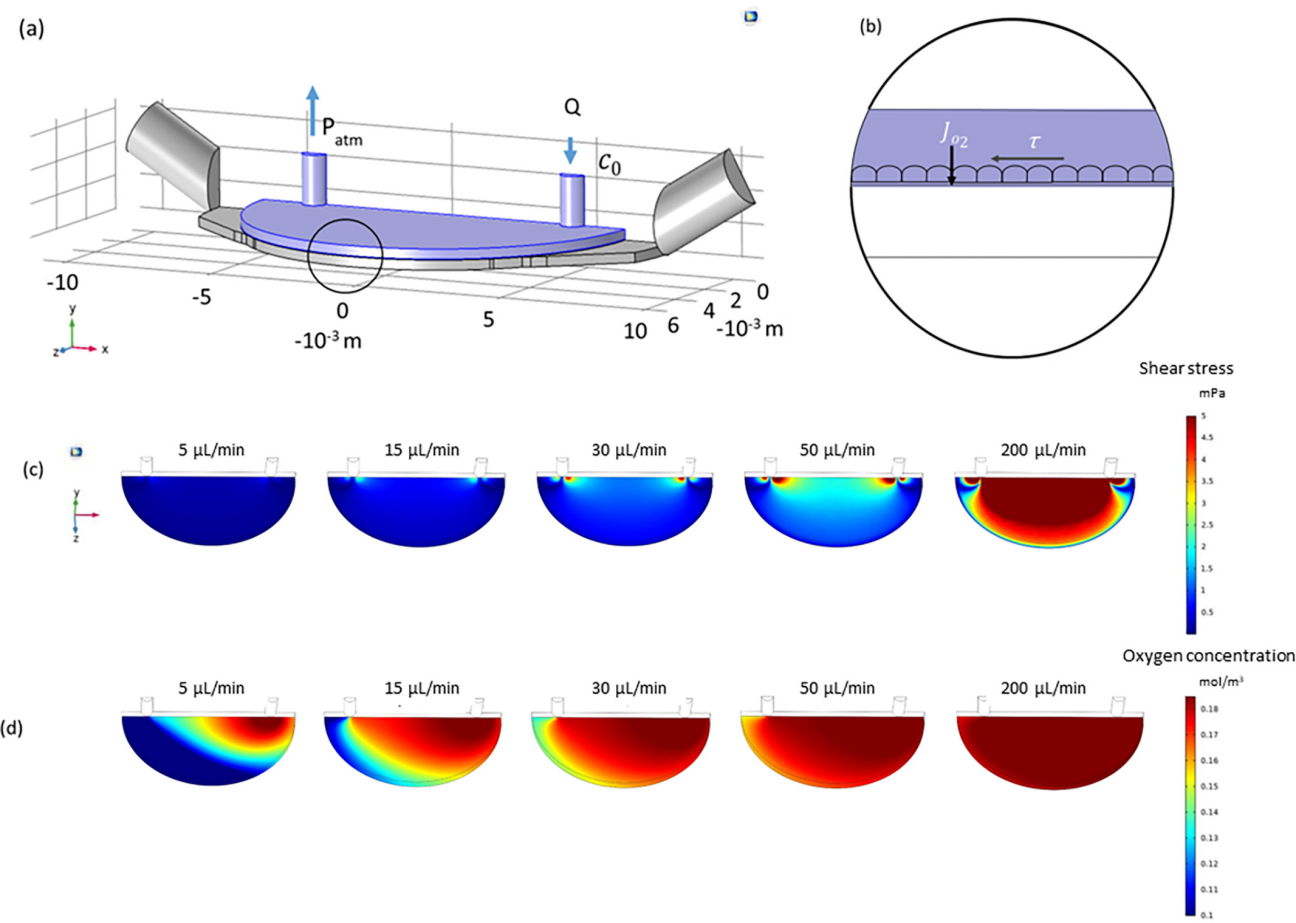
Computational fluid dynamic simulations. (a) The simulations were run on the apical chamber (blue) by solving the Navier–Stokes equation. Different flow rates from 5 to 200 *μ*l/min were tested. (b) To calculate oxygen concentrations, the mass transport equation was implemented setting the oxygen concentration at the inlet and a known, constant oxygen flow rate (J_O2_) was imposed at the membrane. (c) Shear stress at the membrane level considering different flow rates. (d) Oxygen concentration at the membrane level considering different flow rates.

To set the optimal perfusion conditions for Caco-2 cell culture, we made a computational simulation on the MINERVA 2.0 device [Fig. 2(a)], estimating the shear stress (SS) profile and oxygen concentration (OC) at the membrane level [Fig. 2(b)].

The simulation showed averaged SS at the membrane level depending on the inlet velocity (5–200 *μ*l/min) [Fig. 2(c)]. For the perfused samples, we selected the flow rate of 30 *μ*l/min to avoid cellular damage in correspondence with shear stress peaks still guaranteeing an average SS higher than 0.67 mPa (i.e., 0.85 mPa) (Table I), in accordance with the literature.^31^

**TABLE I.** Averaged shear stress at the membrane level considering different flow rates. Shear stress calculated at the membrane level.

Q (*μ*l/min)	5	15	30	50	200
SS (mPa)	0.143	0.426	0.855	1.427	5.709

The oxygen concentration decayed in the direction of perfusion. However, imposing a flow rate of 30 *μ*l/min and a maximum and constant cell consumption, OC remained positive, confirming there was no oxygen deficiency (0.13 mol/m^3^) [Fig. 2(d)].

### Cell viability and cytoarchitecture characterization

**FIG. 3.**
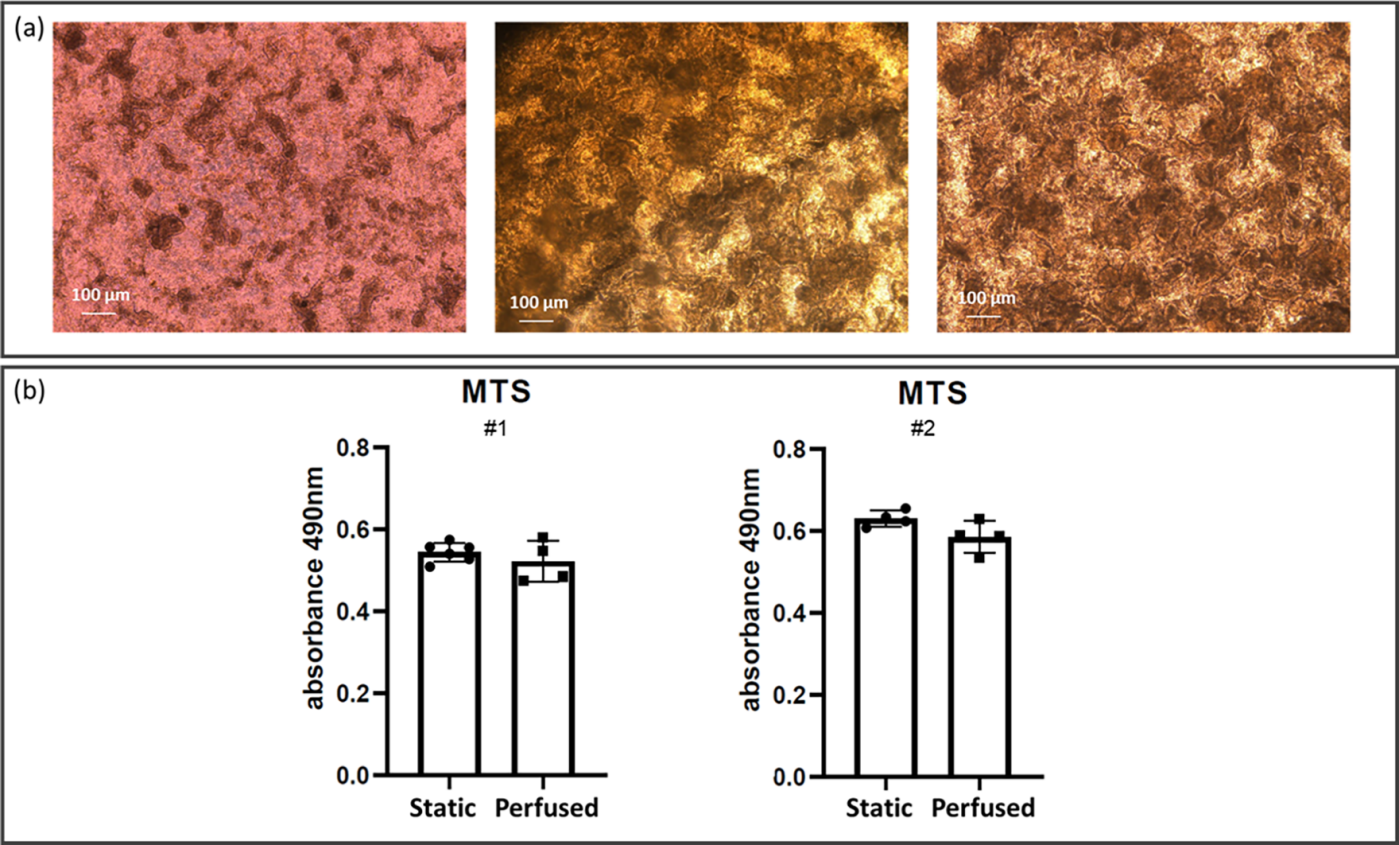
Cell layer differentiation and metabolic activity after 14 days from cell seeding. (a) Phase contrast images of Caco-2 cells cultured in static (left) and in perfused (center and right) conditions. In the center position, an image acquired with the insert still sandwiched in the MINERVA 2.0 device. In the right panel, the same insert after removal from the MINERVA 2.0 device. (b) Cell metabolic activity was assessed with the MTS assay. Two independent experiments were performed. Each experiment involved at least four inserts for both the static and perfused conditions, and was repeated twice (#1, #2). Mann–Whitney U-test showed no significant difference between the two groups (p > 0.05).

During perfusion, Caco-2 cells were monitored daily by light microscopy [Fig. 3(a)]. Phase contrast images on day 7 of dynamic culturing showed the presence of columnar shapes in perfused samples, whereas the controls had more planar distribution [Fig. 3(a)]. Cell metabolic activity assessed with the MTS assay showed no significant differences between the two groups. Comparable results were obtained in two independent experiments [Fig. 3(b)].

**FIG. 4.**
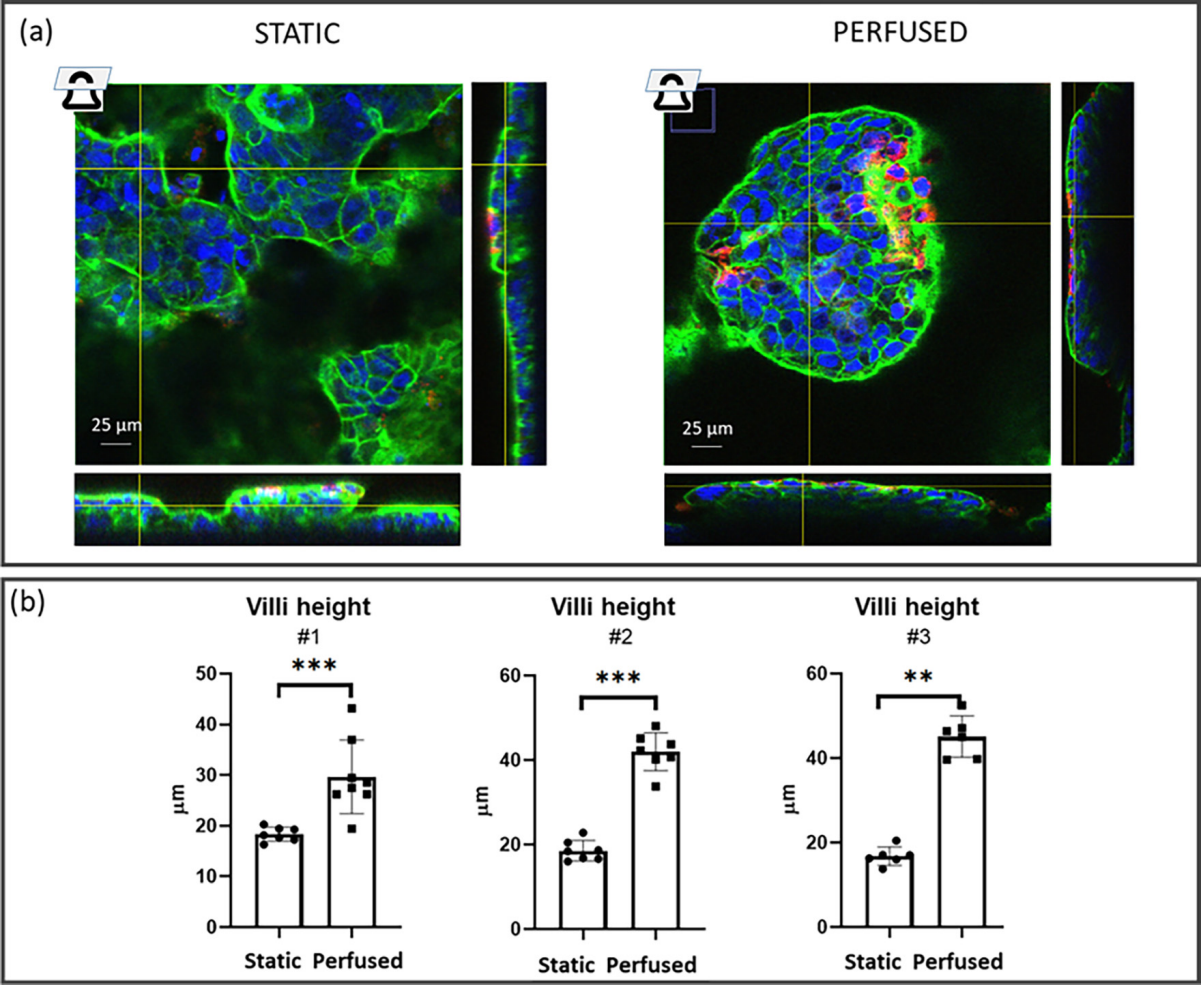
Epithelial height. (a) Epithelial height was calculated using orthogonal views of immunofluorescence images (F-actin in green, DNA in blue, and mucin-2 in red) acquired with a confocal microscope, taking the highest and lowest actin layers in each image. (b) Epithelial height was analyzed on three independent experiments (#1, #2, and #3) for static and perfused samples. For each experiment, at least four different images were acquired. Each image corresponds to a dot in the plot. Mann–Whitney U-test, ^**^p < 0.01; and ^***^p < 0.001.

The immunofluorescence images supported the presence of vertical formations reminiscent of intestinal villi (Fig. 4). The vertical formation height was about 40 and 20 *μ*m in the perfused and static samples, respectively, suggesting higher 3D-structure development likely induced by shear stress (P-value< 0.0001). Comparable results were obtained in three independent experiments [Fig. 4(b)]. The formation of 3D structures seemed more frequent in the central region of the Transwell-like insert than in the peripheral one. This aspect was more evident in the inserts cultured in the MINERVA 2.0 than in the static condition, maybe due to a shear stress gradient.

**FIG. 5.**
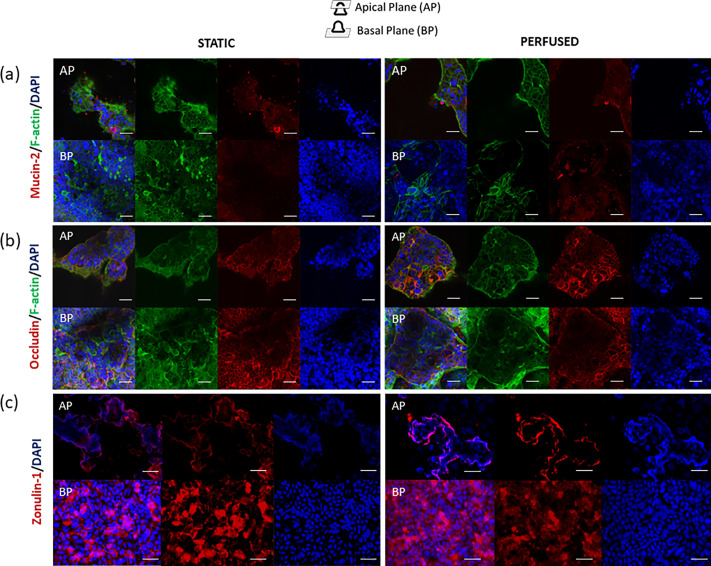
Confocal immunofluorescence images of Caco-2 (left = static; right = perfused) at 14 days from the seeding. (a) F-actin was stained in green, DNA in blue, and mucin-2 in red. (b) F-actin was stained in green, DNA in blue, and occludin in red. (c) the DNA was stained in blue and zonulin-1 in red. The first row corresponds to the apical plane (AP) of the villus, and the row below, to the basal plane (BP). The scale bar corresponds to 25 *μ*m.

To further investigate cell differentiation, we examined the cell shape and polarity with F-actin and mucin-2 staining [Figs. 4 and 5(a)]. Mucin-2 was detected in both perfused and static control groups. Perfused samples gave a discontinuous F-actin immunostaining signal along the villi-like line, while F-actin in static control was homogeneously distributed [Fig. 4(a)].

The ability of Caco-2 to form an efficient cellular epithelial-like barrier in MINERVA 2.0 was investigated by observing the tight junction (TJ) formation at first through occludin expression [Fig. 5(b)].

In both perfused and static conditions, occludin showed a strong fluorescence , indicating no perfusion-induced TJ loss [Fig. 5(b)].

To further evaluate the intestinal barrier TJ function, we also investigated zonulin-1 (ZO-1) expression [Fig. 5(c)]. We found comparable ZO-1 levels in static and perfused samples, thus, confirming no TJ alteration under dynamic conditions.

### Apparent permeability (P_app_) by FITC-dextran and trans-epithelial electrical resistance (TEER)

**FIG. 6.**
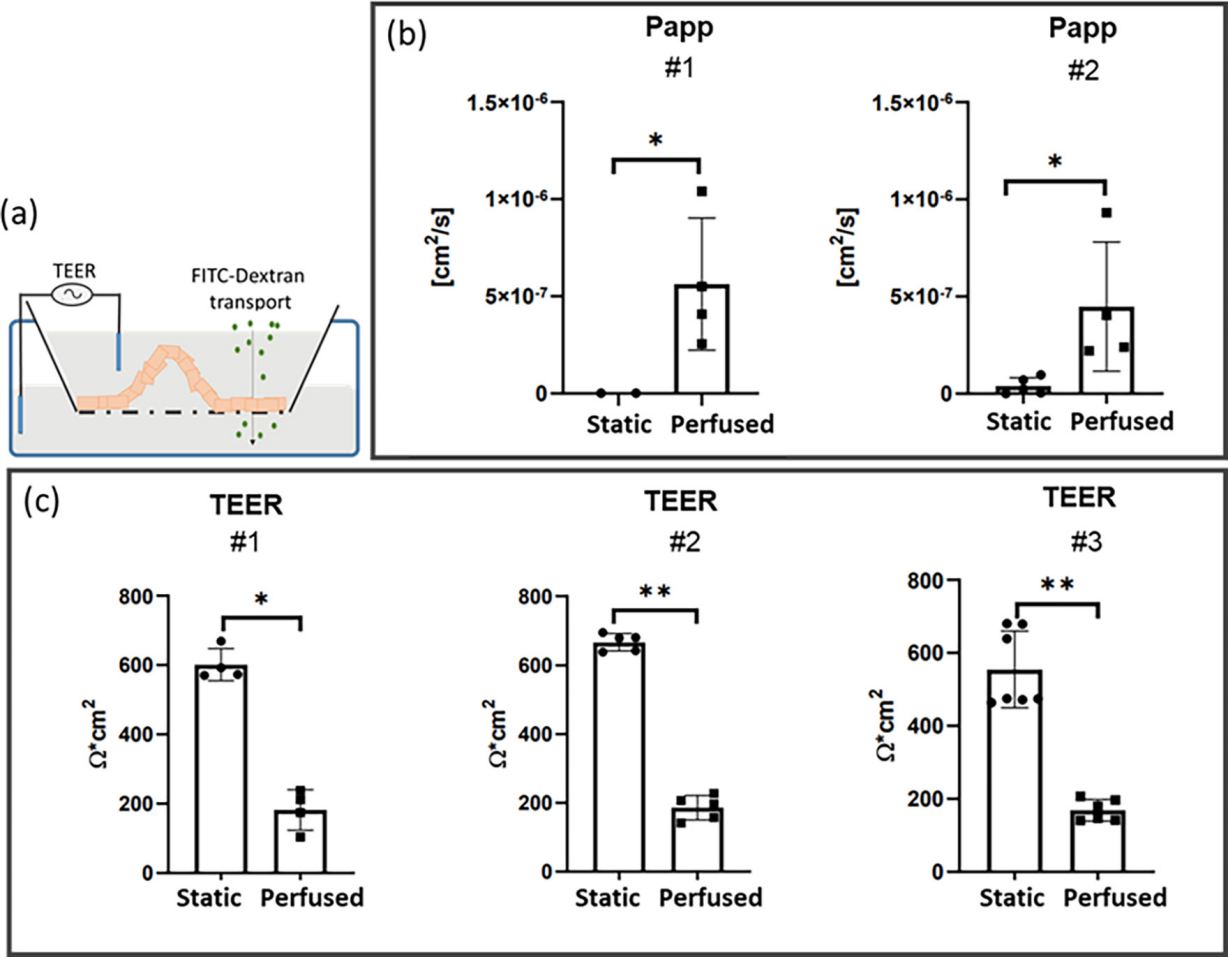
Gut epithelial barrier function. (a) Graphic representation of TEER and FITC-dextran permeability. (b) Apparent permeability (P_app_) was tested twice (#1, #2). Each experiment involved at least four samples for each static and perfused condition. ^*^ = p < 0.05, Mann–Whitney (MW) U-test. (c) TEER was measured three times (#1, #2, and #3). Each experiment involved at least four inserts for each static and perfused condition. ^*^p < 0.05, ^**^p < 0.01, Mann–Whitney U-test.

To evaluate the integrity of the cellular layer, we examined the transport of FITC-dextran across the Caco-2 cell layer and measured the TEER [Fig. 6(a)]. The apparent permeability of FITC-dextran (Papp) increased in the perfused inserts as demonstrated by results from two independent experiments run at least in quadruplicate (p < 0.05) [Fig. 6(b)].

TEER was significantly reduced (p < 0.05). On day 14 from cell seeding, the TEER of the static culture was about 650 Ω cm^2^, compared to around 190 Ω cm^2^ of the perfused culture [Fig. 6(c)]. Coherent data were obtained from the three independent experiments run at least in quadruplicate.

### Caco-2 gene expression under dynamic culturing

**FIG. 7.**
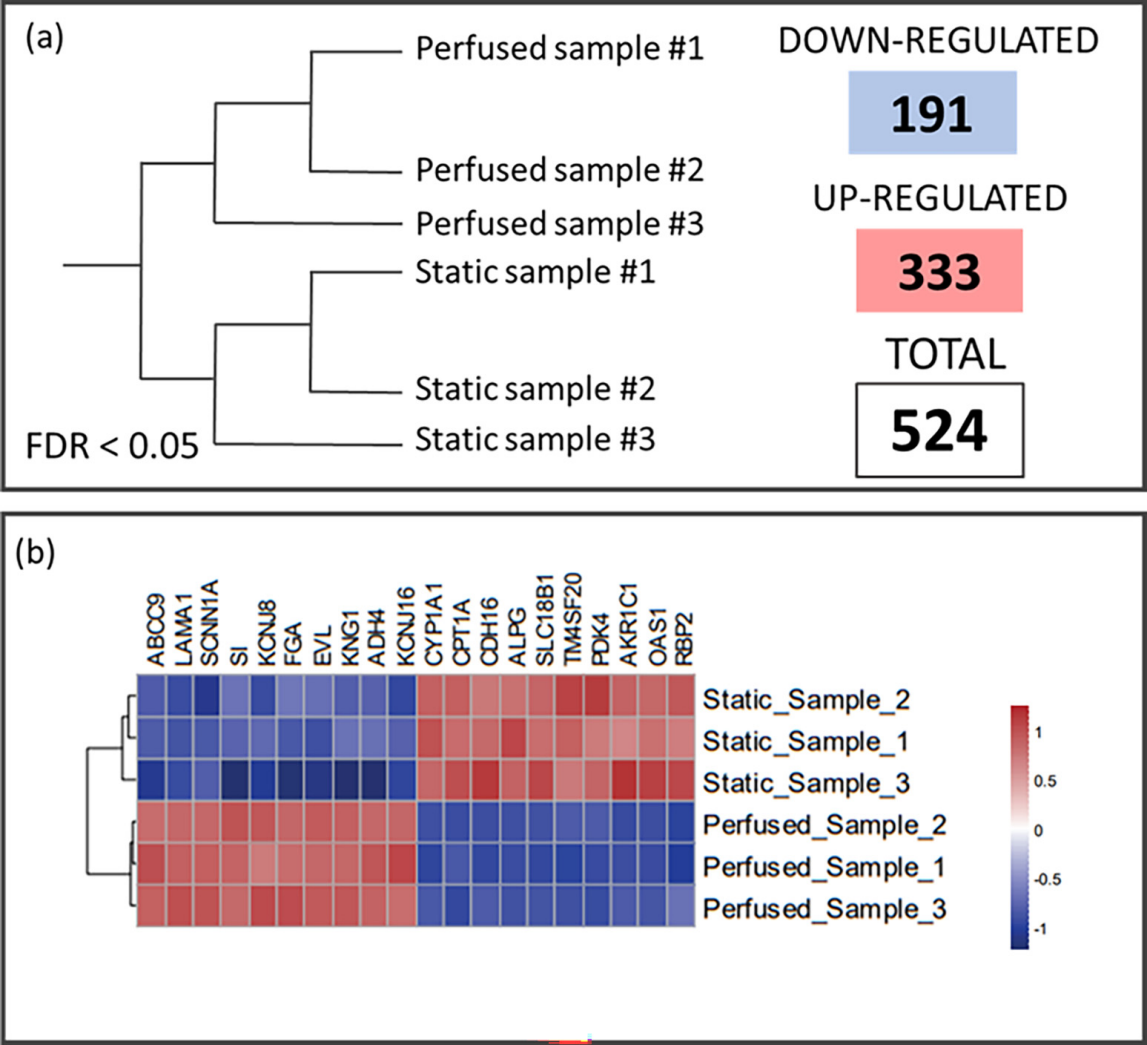
RNA sequencing analysis. (a) Graphic representation of sample clustering based on the gene differential expression. 524 genes were deregulated (333 genes upregulated and 191 genes downregulated), with FDR < 0.05. (b) The heatmap of the ten most significantly downregulated (in blue) and upregulated (in red) genes.

To assess whether dynamic perfusion affected Caco-2 transcriptomic profile, a RNASeq analysis was performed starting from a library generating 60605 potential targets (accession number E-MTAB-11949). A two-step filter was applied to keep only the annotated genes with a reliable level of expression, for a total of 24 309 genes. Compared to the static ones, in the perfused samples, respectively, 333 and 191 genes were upregulated and downregulated. The large number of deregulated genes (524) allowed clear clustering between the dynamic and the static control samples [Fig. 7(a)]. Considering the 10 most significant upregulated and downregulated genes [Fig. 7(b)], there were differences in the genes functionally related to cell–cell\cell–matrix adhesion, ion channels, and metabolism and the development-related genes.

There were 16 pathways upregulated (supplementary material) (macro categories of KEGG: metabolism, ECM formation, vascular endothelial growth factor, autophagy, Tryg, specialized pro-resolving mediators, solute carrier transporters) and four pathways were downregulated (macro categories of KEGG: immune and development, platelet-derived growth factor, scavenging by class H receptors).

### MINERVA 2.0 serial connection

**FIG. 8.**
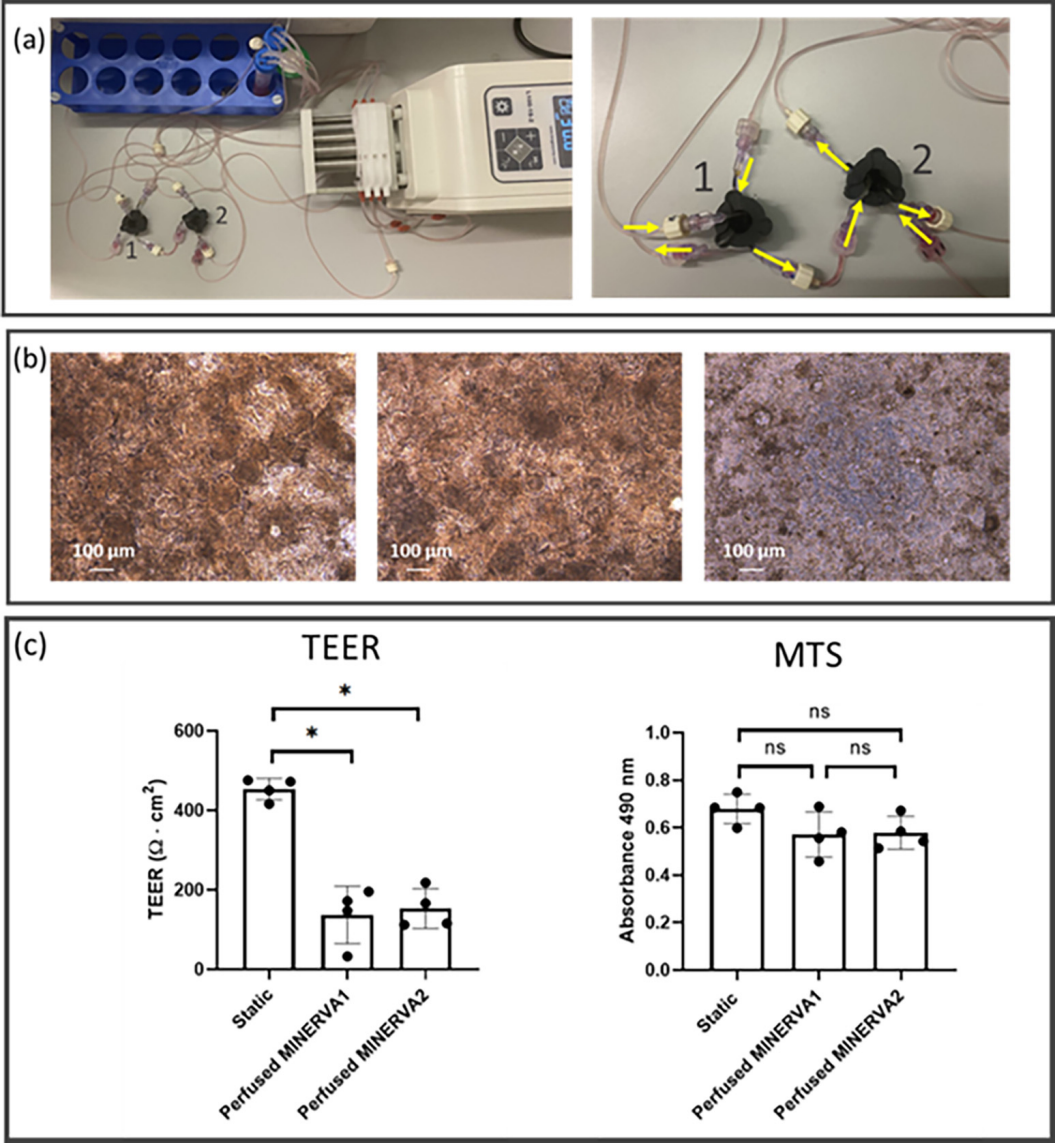
In-series perfusion via Luer-lock connection. (a) View of the MINERVA 2.0 setup for in-series perfusion (left) and magnification of the device connection (right). The inlet and the outlet of the apical chamber of the first device (labeled as 1 in the pictures) are connected to the pump and the reservoir, respectively. The inlet and the outlet of the basal chamber of the first device are connected to the pump and the inlet of the apical chamber of the second device (labeled as 2 in the pictures), respectively. The inlet and the outlet of the apical chamber of the second device are connected to the output of the first device's basal chamber and the reservoir, respectively. The inlet and the outlet of the basal chamber of the second device are connected to the pump and the reservoir, respectively. (b) Phase contrast images of Caco-2 cells cultured in perfused (left and center for first and second connected device, respectively) and in static (right) conditions. (c) TEER and MTS data for static and in-series perfusion. The experiment involved four replicates for each static and in-series perfused condition. ^*^p < 0.05 Kruskal–Wallis test.

With the aim of assessing MINERVA 2.0 performance in the perspective of a multiorgan platform, we tested the suitability of MINERVA 2.0 device to provide physiological stimuli even when connected in series to another MINERVA 2.0 device. During the in-series perfusion [Fig. 8(a)], Caco-2 cells were observed through the optically accessible windows of the devices. Phase contrast images on day 7 of in-series perfusion showed comparable morphological distribution between the first and the second connected device [Fig. 8(b) left and middle panel, respectively]. The static controls confirmed almost planar distribution [Fig. 8(b), right panel]. On day 14 from cell seeding, cell metabolic activity assessed with the colorimetric MTS assay showed no significant differences among the three groups [Fig. 8(c), right panel]. TEER of the static culture was about 450 Ω cm^2^, compared to around 150 Ω cm^2^ of both the perfused samples [Fig. 8(c), left panel].

## DISCUSSION

In the present work, we describe MINERVA 2.0 millifluidic device exploitation as gut-on-a-chip starting from the computational modeling till its biological assessment. We tested MINERVA 2.0 alone and once serially interconnected in a configuration prodromal to address the complexity of multiorgan crosstalk, a current relevant topic in the research field of human diseases, including neurodegenerative and other disorders.^16,35^ In particular, MINERVA 2.0 was designed to: (a) guarantee affordable manufacturing, because it is based on 3D printer techniques; (b) host millimetric culture chambers to culture higher quantity of cells; (c) allow continuous cell monitoring by microcopy, thanks to its optically accessibility; (d) be versatile as it hosts commercial cell culture inserts that apart dimensional constraint may have very customizable features in terms of membrane material and/or pore dimension and density; (e) be user friendly; (f) be modular and easily connected with other devices in a customized manner.

Once assembled, MINERVA 2.0 forms two independent compartments giving high versatility for culturing solutions. This is supported also by a comparison of this work with another manuscript of our group already published.^36^ By comparing the two models, we can appreciate the versatility of MINERVA 2.0 in terms of type of cell culture (e.g., iPSC vs immortalized cells), different use of culture conditions based on the Transwell-like inserts (e.g., on both sides of the membrane, keeping the two fluid paths separated vs on one side of the membrane), cell seeding mode (e.g., cells embedded in a gel vs cells seeded directly on the membrane), and different organ development (e.g., liver vs intestine).^36^ Moreover, the two glass inserts of MINERVA 2.0 in both the culture chambers allow optical access by light transmission or confocal microscopy, a desirable feature as already reported in other solutions.^37,38^

MINERVA 2.0 has an easy-to-use snap-fit closure system combined with an O-ring system to ensure a perfect seal even under flow-induced pressure. The Luer-lock connectors at the inlet and outlet of each chamber can easily connect two or more MINERVA 2.0 devices in series, resulting in versatile multiorgan-on-a-chip configurations that overcome the intrinsic limitations of some currently available body-on-a-chip tools described in the literature that involve a not-customizable preset platform.^21,28^

We developed a novel millifluidic gut-OoC based on human Caco-2 cells frequently used for the same purpose in OoC.^6,22,23,39^ To ensure the optimal perfusion condition, we first implemented computational simulations to select the range of perfusion parameters to reproduce the shear stress (SS) and the oxygen supply (OS) required for gut cell differentiation and possible villi-like structure formation. We selected the lowest flow rate able to guarantee the average shear stress reported to support this differentiation effect.^31^

The MTS experimental results gave a confirmation of the suitability of our computational data, with comparable Caco-2 cell viability between static and perfused condition. Moreover, MTS results of in-series perfusion showed no viability differences between the perfused samples and the static ones supporting the modularity of the developed device. In line with this, phase contrast images confirmed enhanced 3D layer development in both single and in-series perfusion. In single perfusion, we also investigated the Caco-2 layer morphology in immunofluorescence studies. Morphological analysis showed differences in 3D cytoarchitecture. Our results support the role of perfusion in inducing Caco-2 cells to polarize and form villi-like structures twofold taller than the cells cultured in static condition (40 and 20 *μ*m for perfused and static samples, respectively).^23,40^ In accordance with this, cells in the static samples tended to be organized in cell agglomeration [darker spots in Fig. 3(a), on the left], probably a step prodromal to villi development.^29^ The villi-like structures increased from the periphery to the central part of the Transwell-like membrane, and this phenomena may be related to a shear stress (SS) gradient effect, as the calculated SS profile is not perfectly uniform along the porous membrane. Nevertheless, it is worth noting that at 30 *μ*l/min, less of 10% of the whole area is subjected to SS higher than 2 mPa. To get firmer conclusions, this qualitative observation should be better investigated with a quantitative approach.

We noticed in dynamic condition the presence of mucin-2, a mucoprotein commonly found in the human intestine,^33^ also at the villous structure apical plane. This desirable physiologic feature confirms the maintenance of this key cellular function under perfusion too.^32,40,41^ Mucin-2 apical presence was consistent with the mucoprotein deposition *in vivo*.^22,42^ Also, at the plasma membrane, both the controls and the perfused samples showed occludin expression.^43^ Occludin presence in TJ holds great relevance as it is functionally involved in villi dynamics. Data on the *in vivo* normal juvenile intestine reports that TJ increases in tightness from the basal portion or crypt to the villus tip.^44^ This is probably induced to guarantee the cell migration up to the apical plane required to replace the cells that are lost during cell sloughing.^29,45,46^ Cell sloughing indicates the process of cells that, generated from stem cells located in crypts between the villi, migrate from the crypt up the villus, differentiate, and finally undergo apoptosis and are sloughed off from the villus tip. Sloughing process duration is compatible with the timing of experiments carried out with the MINERVA 2.0 device. This confers to the system the capability to induce the growth of an intestinal cell construct with dynamic cellular movements resulting suitable also to get insight into the physiological cell sloughing process.^47–49^

The absence of difference observed in terms of zonulin-1 expression between static and perfused samples further confirmed physiological barrier expression in MINERVA 2.0 device, a desirable feature for intestinal permeability modulation.^5,50^

To this respect, the permeability of FITC-dextran molecules showed enhanced P_app_ induced by the perfusion, and thus greater molecular passage through the cell layer. After 7 days of perfusion, the TEER decreased, reaching a value coherent with *in vivo* small intestine data.^51,52^ Comparable results were obtained in in-series perfusion confirming the MINERVA 2.0 suitability to modular assembly at increasing complexity. Since we did not notice any sign of layer disruption in the perfused samples, the lower TEER and higher P_app_ may be induced by the presence of the columnar shape or/and by a lower cellular differentiation at the basal plane.^31,45^ To definitely exclude the cell layer damage induced by Transwell-like insert extraction, as next step, we will design integrated electrodes in MINERVA 2.0, as in the study by Cacopardo *et al.* However, in the latter study, Cacopardo *et al.* observed TEER increment after 7 days of perfusion, but the start of perfusion 24 h after seeding does not allow direct comparison with our system.^43^

To fully evaluate the biological impact of dynamic culturing in our device, we performed an unbiased transcriptome analysis (RNASeq). A first important finding was a significant downregulation in the perfused CaCo-2 cells of the ion channels and related ATP-binding cassette (*SCNN1A, KCNJ8, KCNJ16*, and *ABCC9* gene), key modulators of TJ-related permeability functions.^53^ This aspect suggested that also TJ function may be changed by the dynamic condition at molecular level. In general, the RNASeq results clearly showed clustering of the perfused and static samples, with deregulation of 524 genes, supporting the appreciable impact of perfusion. Among the ten most significant up- or downregulated genes there was strong deregulation of cell–cell and cell–matrix interaction genes, such as cytoskeletal components, cytoskeleton-related genes, extracellular matrix, and mechanotransduction-related genes (*FGA, EVL, LAMA1, CDH16*, and *TM4SF20* gene), as also evident from the upregulation of the fibronectin matrix formation pathway (supplementary material).

The RNASeq results confirmed the strong influence of the flow rate on the development of the cellular layer, on cell proliferation (*RBP2,*^54^ *KNG1*^55^ gene) and on epithelial cell polarity.^56,57^ The higher expression of *TM4SF20* (transmembrane 4L six family member 20, a gene involved in cell proliferation, motility, and cell adhesion), of *CPTA1* (carnitine palmitoyltransferase 1A, which can promote cancer cell proliferation), and of *PDK4* (pyruvate dehydrogenase kinase 4, a gene that influences cell proliferation) supports the effects of perfusion in stimulating growth, reproduction, and differentiation of the Caco-2 cells. All these dynamic cell features might also impact the energy consumption and fatty acid metabolism, which were deregulated by pathway analysis (KEGG categories “metabolism” and “triglyceride metabolism pathway”) (supplementary material).^58–61^

In line with a polarization and differentiation effect supported by morphological analysis, the laminin subunit α1 (*LMNA1*) gene was downregulated in perfused conditions compared to the control.^39^ The laminin subunit α1 has been reported to drop in mature epithelial tissues. In line with this, laminin subunit α1 downregulation in the perfused condition supports the MINERVA 2.0 as suitable tool to provide more favorable conditions for the polarization and maturation of the epithelium.^39^

The promotion of a mature intestine epithelium layer in MINERVA 2.0 is supported even more by the upregulation of the retinol-binding protein 2 (*RBP2*) gene, usually expressed in active absorptive cells of the proximal small intestine.^62^ Coherently with villous structure height observed in the immunofluorescence assay, sucrase-isomaltase (SI) was downregulated in perfused samples. SI is the gene essential for the digestion of dietary carbohydrates and reaches its highest level in the lower and mid-villi.^63^ The downregulation of SI suggests some parallelism with endothelial cells, which after SS show reduced glucose uptake.^64^ However, we did not measure glucose metabolism here, and further research is needed to confirm this observation.

To sum up the biological data on Caco-2 cells in MINERVA 2.0, perfusion stimulates the Caco-2 cells to develop polarized columnar epithelial cells whose cytoarchitecture was reminiscent of villous structures, with the presence of actin, mucin-2, occludin and zonulin-1 markers. The higher P_app_ and lower TEER may be the result of higher and dynamic endothelial structures.^65^ This hypothesis may be in accordance with the literature supporting a negative linear correlation between TEER and the epithelial height in well-differentiated cultures.^45,66,67^ Additionally, on day 14 from the cell seeding, when perfused samples showed a better development, the static ones started organizing in cell agglomerations, likely villi prodromes. At this time, we observed small villi-like and multilayer formations in the static samples, in accordance with reports of Caco-2 layer full development around 21 days of culture.^68^ In this sense, the dynamic condition accelerated an intrinsic potential differentiation of Caco-2 line and certainly affects their gene expression pattern.

### Conclusion

We have reported MINERVA 2.0 millifluidic OoC device and its application as gut-on-a-chip. MINERVA 2.0 guarantees optimal flow rate conditions for Caco-2 cells, also when two devices were connected in a serial configuration. Caco-2 reacted to perfusion with robust changes in gene expression, three-dimensional cell layer production characterized by TJ expression, and TEER values similar to the *in vivo* human intestinal barrier.^51,52^ Overall, this application of MINERVA 2.0 moves forward its exploitations in OoC and opens the way for further customization and biologic assessment in biological systems/organs other than the gut.

## METHODS

### Millifluidic device

MINERVA 2.0 (IT Patent n. 102019000016376) represents the next generation of our previously described MINERVA 1.0 device.^34^ In particular, MINERVA 2.0 was designed to host one sample instead of three samples integrated in a single rectangular device, guaranteeing more versatility. Moreover, MINERVA 2.0 was designed to be compatible with commercial cell culture inserts instead of being equipped with a custom-made cell culture insert, allowing the direct comparison with standard static cell culture. MINERVA 2.0 device was 3D printed in nylon by a multi-jet fusion technique. The 3D printed parts (one basal component and one apical component) were then equipped with Tygon laboratory tubings (Qosina) for flow perfusion and Luer-locks (Qosina) and round-shaped glass microscope slides for optical accessibility (Menzel Glaser, VWR). The complete system consisted in the superior apical part fitting a permeable cell culture insert that was hosted in the inferior basal part. This plug-and-play approach enabled the sealing of the system forming two fluidic hemi chambers interfaced through the permeable porous membrane. The devices were sterilized by UV rays (SafeMate cabinet) for 10h or with hydrogen peroxide (V-PRO^®^ 60 Low Temperature Sterilization System).

### Numerical evaluation of MINERVA 2.0

To assess the suitability of MINERVA 2.0 millifluidic devices for dynamic culture of human intestinal Caco-2 cells, we ran multiphysics computational simulations (computational fluid dynamics and mass transport analysis) with the software COMSOL Multiphysics^®^ (Burlington, MA, USA). We ran the simulation in the worst-case scenario where cells are oxygenated only from the apical chamber [Figs. 2(a) and 2(b)]. The geometry of the MINERVA 2.0 apical chamber was extracted from the internal space of the culture chamber using Solidworks^®^ software. Numerical simulations were implemented in the apical chamber to estimate the optimal flow rate in terms of oxygen supply and SS profile at the membrane level in correspondence to the cellular layer. Different flow rates were tested, from 5 to 200 *μ*l/min (i.e., 5–15–30–50–200 *μ*l/min). Table II lists the numerical parameters used for the simulation.

**TABLE II.** Parameters for computational simulation. Values and properties set for computational fluid dynamic simulation.

Properties	Values
Inlet velocity	1.67 × 10^−4^–667 × 10^−3^ m/s
Inlet oxygen concentration	0.195 mol/m^3^
Outlet pressure	0 Pa
Oxygen diffusion coefficient	2.00 × 10^−9^ m^2^/s
Dynamic viscosity of culture medium at 37 °C (Ref. 70)	1.023 × 10^−3 ^Pa s
Density of culture medium at 37 °C	1000 kg/m^3^
Oxygen consumption (outlet flow at the interface)	7.34 × 10^−8 ^mol/(m^2^ s)
Condition at the wall	No-slip condition

Fluid velocity vector u was determined by the Navier–Stokes equation in the stationary condition,

$$\rho \left(u\cdot \nabla \right)u=\nabla \cdot \left[-pI+K\right]+F$$

and mass-balance equation,

ρ∇·u=0,

where ρ is the fluid density, u is the velocity vector, p is the fluid pressure, I is the identity matrix, and F is the volume force vector. K is the viscosity tensor defined as

$$K=\mu (\nabla u+{\left(\nabla u\right)}^{T}),$$

where μ is the medium dynamic viscosity.

Shear stress was calculated at the membrane as follows:

$$\tau =-\mu (\frac{\delta u_{x}}{\delta z}),$$

where *u_x_* is the velocity component vector parallel to the perfusion direction, and z is the direction perpendicular to the basal plane. To assess the suitability of the device for Caco-2 cell culture, we averaged SS values at the membrane level and compared them with the literature data.

We estimated oxygen distribution with the equation of mass transport of diluted species,

$$\nabla \cdot (-D_{i}\cdot \nabla c_{i})+u\cdot \nabla c_{i}=R_{i},$$

where the reaction term was set null ( $R_{i}$ = 0) in the control volume, and $D_{i}$ is the oxygen diffusion coefficient in the medium.

As boundary conditions, we set the inlet oxygen concentration as shown in Table II and constant oxygen flux at the interface with the cells [Fig. 2(b)]. All the device walls and the membrane were imposed as “wall” condition. We set the condition as the worst-case scenario, assuming maximum cell density from day 0. Considering homogeneous cell distribution throughout the membrane with the initial cell density of 5 × 10^4^ $\text{cells}/{\mathrm{cm}}^{2}\frac{}{}$ doubling in 80 h and basal oxygen consumption^69^ of $2.1\;\;n\;\text{mol}(O_{2})/\text{min}10^{6}\text{cells}$, the constant oxygen flow was estimated as follows:

$$J=2.1\;\frac{\text{nmol}(O_{2})}{\text{min}10^{6}\;\text{cells}}\times 5\times 10^{4}\frac{\text{cells}}{{\mathrm{cm}}^{2}}\times 2^{d}\times \frac{\;\text{min}}{60\;s}\times \frac{\;{\mathrm{cm}}^{2}}{10^{-4}\;m^{2}}=7.34\times 10^{-8}\frac{\text{mol}\left(O_{2}\right)}{s\;\;m^{2}},$$

where doubling *d* is 4.2, considering 14 days of perfusion and 80 h of cell division time.

### Caco-2 cell model

Human intestinal cell line (Caco-2 cells, ATCC^®^ HTB-37) was cultured in high glucose Dulbecco's modified Eagle's medium (DMEM) (Gibco), supplemented with 20% of heat-inactivated fetal bovine serum (Gibco), 2 mM L-glutamine (Euroclone), and 100 units/ml of penicillin and 100 *μ*g/ml of streptomycin (Euroclone). Caco-2 cells between passages 30 and 40 were seeded at the density of 5 × 10^4^ cells/cm^2^ on pH equilibrated PET membrane, with a surface area of 1.1312 cm^2^, pore diameter of 0.4 *μ*m, and density of 2 × 10^6^ pores/cm^2^ (Greiner Bio-One), and coated with 30 *μ*g/ml collagen (Sigma-Aldrich) according to the specification in the datasheet. All the culture inserts were incubated at 37 °C and 5% CO_2_ for 7 days with medium renewal every 2 days.

### Perfused cell cultures

After 7 days in static conditions, the cell-seeded Transwell-like inserts were randomly split into two groups: the static samples and perfused ones. The static samples were maintained in wells for other 7 days, with medium renewal every 2 days. We refer to these samples as “static samples” and represent the control. The other group of the cell-seeded Transwell-like inserts were hosted in the respective devices. Each device was connected to a reservoir with five connections: two for medium inlets, two for medium outlets, and one for a filter connection to guarantee no pressure variation in the reservoir. Both the hemi-chambers were perfused using peristaltic pumps (Longer Precision Pump Co.) at 30 *μ*l/min. We refer to these as “perfused samples.”

### Cell viability

Human gut Caco-2 epithelial cells were seeded in Transwell-like inserts and cultured in static conditions for 7 days before being positioned in the MINERVA 2.0 devices and perfused at 30 *μ*L/min for another 7 days. To assess cell viability, the MTS assay (CellTiter 96^®^ Aqueous One Solution Reagent from Promega) was done on perfused and static samples. Cells were incubated for 1 h at 37 °C and 5% CO_2_ with 0.5 ml of MTS solution in complete medium (158 *μ*g/ml MTS) in the apical chamber. Then 100 *μ*l of solution was transferred from the apical compartment to a 96-well plate, and the UV-absorbance of the formazan crystals was measured at 490 nm (Tecan Spark 10 M). The measurement was repeated twice for each sample.

### Biological characterization by immunofluorescence assay and confocal microscopy: villi, mucin 2, and tight junctions

After removing the samples from the MINERVA 2.0, they were rinsed twice with PBS with Ca^2+^ and Mg^2+^ for 5 min and fixed for 40 min in warmed paraformaldehyde (4% PAF). Samples were then washed three times for 5 min in PBS. To block nonspecific binding of antibodies, 300 *μ*l of blocking solution (0.25% Triton-X-100, 4% NGS in PBS) was added to each sample for 1h at RT under stirring. Samples were incubated overnight at 4 °C with primary antibodies diluted in PBS with Triton-X-100 0.25% and NGS 1%.

To confirm gut barrier function, we investigated occludin and zonulin-1 (ZO-1) expression following the same protocol. To visualize occludin, we used mouse anti-occludin monoclonal antibody (Invitrogen) diluted 1:100; cell function was tested using mouse anti-mucin 2 antibody (Sigma-Aldrich) diluted 1:250; cell polarity was assessed using FITC-phalloidin dye (Sigma-Aldrich) diluted 1:40. For ZO-1, we used rabbit anti-ZO-1 polyclonal antibody (Invitrogen) diluted 1:50.

The next day, samples were rinsed three times for 5 min in PBS and incubated with the Alexa Fluor 647 goat anti-mouse IgG secondary antibody (Jackson IR) diluted 1:750 and Alexa Fluor 647 goat anti-rabbit IgG secondary antibody (Jackson IR) for occludin and ZO-1, respectively, at RT for 45 min in the dark with stirring. After three samples rinses (5 min in PBS), cell nuclei were labeled with Hoechst 33342 (Thermofisher) diluted 1:12 000 for 10 min at RT. Finally, samples were rinsed two more times with PBS before removing the membranes from inserts and mounting them with a drop of FluorSave reagent (EDM Millipore) on microscope slides.

Fluorescence images were acquired either with two confocal microscopes: the Nikon AR1+ (equipped with a 40X water immersion objective with 1.15 N.A. and 0.60 W.D., and a 60X oil immersion objective, with 1.4 N.A. 0.13W.D.) and with an Olympus Fluoview (equipped with a 60× water immersion objective, 1.2 N.A. and 0.28 W.D.). The pinhole was set at 1 Airy Unit. Sample groups were imaged by z-stack acquisitions aiming at covering the full length of the epithelium layer with a maximum step of 0.5 μm. Each image is composed by 1024x1024 pixel^2^.

The images were processed using the open-source software ImageJ (https://imagej.nih.gov/ij/index.html, USA).

To calculate the columnar height, the orthogonal projections (xy and xz) of the z-stacks were run for each image. Height was determined using the F-actin signal estimating the distance between the F-actin distribution at the epithelium base and that at the tip.

### Trans-epithelial electrical resistance (TEER)

TEER was measured on days 7 and 14 from cell seeding. TEER was measured using EVOM (World Precision Instruments, USA) coupled with a chopstick-like electrode. Cell layer resistance (R_measured_; Ω) was calculated placing the shorter electrode in the apical compartment of the inserts and the longer one in contact with the plate. TEER (Ω $\;{\mathrm{cm}}^{2})$ was calculated as follows:

$${\mathrm{TEER}}_{\text{layer}}=\left(R_{\text{measured}}-R_{\text{blank}}\right)\cdot \mathrm{Membrane}\;\mathrm{Area},$$

where R_blank_ was measured on collagen-coated inserts without cells, and the MembraneArea was 1.131 cm^2^. For each sample, we averaged three measures.

### Apparent permeability (P_app_) by FITC-dextran

Barrier permeability was assessed using 4 kDa FITC-dextran (TdB Labs). After rinsing the layers with PBS supplemented with Ca^2+^ and Mg^2+^, 750 *μ*l of culture medium enriched with 1 mg/ml dextran-FITC was added in the apical compartment, and 750 *μ*l of culture medium was added in the basal one. An empty coated Transwell-like insert was used as positive control. Samples were incubated for 3.5 h at 37 °C. After pipetting the medium in the basal compartment, 100 *μ*l of the basal solution was transferred to a black 96-well plate. Each sample was measured twice. The calibration curve was prepared by serial dilutions from 50 to 0.78 *μ*g/ml. To quantify the amount of FITC-dextran transported through the cell layer, fluorescence intensity (488 nm excitation/520 nm emission) from the basal compartment was measured with Tecan Spark 10 M. Each value was subtracted to the blank corresponding to the basal fluorescence of fresh medium.

Apparent permeability (cm s^−1^) was then calculated as follows:^71^

$$P_{\text{app}}=\frac{\frac{dQ}{dt}}{C_{0}\cdot A},$$

where dQ is the fluorescence measured in the basal compartment (*μ*mol), dt is the incubation time (12 600 s); $C_{0}$ is the initial FITC-dextran concentration in the apical compartment (1 mg/ml), and A is the nominal surface area of the membrane (1.131 cm^2^).

### RNAseq

Cells were lysed directly in the Transwell-like inserts with 750 *μ*l of QIAzol lysis reagent (Qiagen). RNA was isolated using an RNeasy Mini Kit (Qiagen). The NanoDrop ND-1000 spectrophotometer (NanoDrop Technologies, USA) was used to measure the concentration of the extracted RNA. According to the TruSeq Stranded Total RNA (Illumina) protocol, 250 ng of RNA for each sample with RIN between 2 and 9 was taken to sequence. Final libraries that give quality and quantity criteria were run on the NextSeq 500 sequencer (Illumina) using a 1 × 75 high-output flow cell with 14 samples/run. FastQ files were generated from raw sequencing reads via Illumina bcl2fastq2. Sequence alignments of total-RNA (stranded) to the reference human genome (GRCh38) were done using STAR (v2.7.4a) in two-pass mode. Raw counts per gene were imported in the R-statistical environment. For RNA-Seq analysis, we used the DESeq2 (v1.28.1) pipeline. Samples were adjusted for library size and counts were transformed using variance stabilizing transformation.

For differential gene expression, we filtered out the genes for which there was less than a single read mapped in the sum of all samples and retained only the genes expressed at a reliable level (according to the DESeq Independent-filtering procedure). We considered genes differentially expressed with |log2foldChange (FC)|≥1 and an adjusted P value ≤ 0.05, where |log2(FC)|is the ratio between perfused and static samples, and the adjusted P value corresponds to the P value adjusted according to the false discovery rate (FDR).

The heatmap was obtained by VST (variance-stabilizing transformation), and we used the clustering method ward.D2 (distance “Euclidean” clustering and distance “correlation” for column and row, respectively).

### MINERVA 2.0 modularity

After 7 days in static conditions, the cell-seeded Transwell-like inserts were hosted in MINERVA 2.0 devices. Two MINERVA 2.0 devices were connected in series according to the MINERVA platform requirement, where the interaction among cells in different devices must be mediated by the membrane to avoid cross-contamination of cells. In particular, the apical chamber of the second device was perfused by the hydraulic connection with the basal chamber of the first one [Fig. 8(a)]. The inlet and the outlet of the apical chamber of the first device are connected to the pump and the reservoir, respectively. The inlet and the outlet of the basal chamber of the first device are connected to the pump and the inlet of the apical chamber of the second device, respectively. The inlet and the outlet of the apical chamber of the second device are connected to the output of the first device's basal chamber and the reservoir, respectively. The inlet and the outlet of the basal chamber of the second device are connected to the pump and the reservoir, respectively. The devices were perfused using peristaltic pumps (Longer Precision Pump Co.) at 30 *μ*l/min for 7 days. We refer to these samples as “in-series-perfused samples.” Four replicates were performed.

After 7 days of perfusion, the perfused inserts were removed and were analyzed in terms of morphology, TEER, and MTS assay. As controls, four samples were maintained in static conditions for 14 days from the seeding day, with medium renewal every 2 days. We refer to these samples as “Static samples.”

### Statistical analysis

For biological experiments, results are reported as mean ± standard deviation (SD). For each test, we ran at least two independent experiments each with at least four replicates for both the perfused and static control groups. We analyzed all the data with GraphPad Prism software (GraphPad Software, Inc.). Statistical analysis was done in GraphPad Prism (La Jolla, California, USA), with Mann Whitney U-test to determine differences between the perfused and static groups. Kruskal–Wallis test was used to compare in-series connected devices with static control. P < 0.05 was considered statistically significant.

## SUPPLEMENTARY MATERIAL

See the supplementary material for RNA sequencing pathway analysis and comparison between static and perfused samples.

## Data Availability

The data that support the findings of this study are openly available in Marco Bolis at https://www.ebi.ac.uk/biostudies/arrayexpress/studies/E-MTAB-11949, Ref. 72.
